# Immunomodulatory and Antibacterial Properties of the Chumash Medicinal Plant *Trichostema lanatum*

**DOI:** 10.3390/medicines5020025

**Published:** 2018-03-21

**Authors:** Matthew C. Fleming, Victoria Hester, Brittany J. Allison, Majie C. Foster, Donna Nofziger, P. Matthew Joyner

**Affiliations:** Natural Science Division, Pepperdine University, 24255 Pacific Coast Highway, Malibu, CA 90263, USA; mcflemin7@gmail.com (M.C.F.); victoriajh0493@gmail.com (V.H.); bjallison@ucdavis.edu (B.J.A.); mcf106@georgetown.edu (M.C.F.); Donna.Nofziger.Plank@pepperdine.edu (D.N.)

**Keywords:** *Trichostema lanatum*, immunomodulatory, antibacterial, macrophage, TNF-α, traditional medicines of North America

## Abstract

**Background:** The woody shrub *Trichostema lanatum* Benth. (Lamiaceae) is native to Southern California and was reportedly used by the Chumash people as a disinfectant and for the treatment of rheumatism. Based on its traditional uses, this study investigated the antibacterial and immunomodulatory properties of extracts from *T. lanatum.* **Methods:** A methanolic extract of the leaves and stems of *T. lanatum* was tested for immunomodulatory activity by measuring the proliferation of murine macrophage cell cultures and the production of the pro-inflammatory cytokine TNF-α by the cells after treatment with *T. lanatum*. Antibacterial activity of the extract against a panel of six Gram-positive bacteria and two Gram-negative bacteria was evaluated using a disc-diffusion assay. **Results:** The *T. lanatum* extract inhibited the growth of Gram-positive bacteria, but not Gram-negative bacteria. Treatment of activated macrophage cell cultures with *T. lanatum* extract resulted in decreased proliferation of the activated macrophages and a decrease in the production of TNF-α. **Conclusions:** These results provide the first pharmacological support for the traditional use of *T. lanatum* by the Chumash people of Southern California as a disinfectant and treatment for rheumatism.

## 1. Introduction

Traditional medicinal plants have historically been an important source for drug discovery [1,2]. In spite of this historical success, there are still large gaps in the existing knowledge of the chemical composition and pharmacological activities of many plants that have been used for medicinal purposes by native populations in the United States. As a designated biodiversity hotspot, the coastal region of Southern California possesses more than 2000 endemic plant species that represent 0.5% of all known plant species on the planet and more than 4000 total plant species [3]. Among this diverse plant population, at least 40 plant species have reported uses as medicines by native people groups, such as the Chumash [4]. Unfortunately, although there is extensive documentation of many medicinal uses of plants by the Chumash Native Americans [4], there are almost no reports of any experimental investigation of the pharmacological effects of these plants. The woody shrub *Trichostema lanatum* Benth. (Lamiaceae) was reportedly used by the Chumash people as a disinfectant and for the treatment of rheumatism [4]. Unfortunately, the traditional methods of preparations of medicines from this plant were not reported. This traditional use as a disinfectant suggests that extracts of this plant may possess antibacterial activity. The term rheumatism often encompasses a wide variety of underlying diseases, but this traditional use suggests that extracts of this plant may possess immunomodulatory pharmacological activity since inflammation and other pathways mediated by macrophages are implicated in many rheumatic diseases [5,6]. Macrophages are specialized cells that express many receptors at their cell surfaces that allow them to rapidly respond to the presence of pathogens and foreign debris in tissue [7]. Because of their central role in both the innate and adaptive immune responses to damage and infection [8,9], macrophages are an excellent model system for the investigation of treatments that modulate the immune response.

Medicinal plants that modulate macrophage cytokine production are an important focus of inflammation research [10,11]. The cytokine TNF-α is responsible for the regulation of pro-inflammatory responses including cytokine recruitment, apoptosis of cells, and phagocytosis of pathogens [8,12]. Uncontrolled TNF-α production can induce sepsis, tissue injury, and long term inflammation [8,12]. Because of its central role in the regulation of inflammation, modulation of the production of TNF-α by macrophage cells may play an important role in the use of plant natural products as medicines.

Very few studies of the chemistry of plants in the *Trichostema* genus have been published [13,14,15] and a search of the literature also found no reports of any pharmacological studies of preparations from plants in this genus. Here, we report the immunomodulatory and antibacterial activities of a methanolic extract from *T. lanatum*. The extract was tested for its ability to alter the production of TNF-α by murine macrophage cells and for inhibitory activity against a panel of both Gram-positive and Gram-negative bacteria. Both anti-bacterial and anti-inflammatory activities were observed, and several phenolic compounds were putatively identified in the extract that are analogues of flavonoids with known anti-inflammatory activity.

## 2. Materials and Methods

### 2.1. General Methods

RAW 264.7 cells were purchased from the American Type Culture Collection (ATCC TIB-71). Live bacterial cultures of Corynebacterium xerosis, Enterococcus faecalis, Bacillus subtilis, Staphylococcus epidermidis, Bacillus megaterium, Staphylococcus aureus, Escherichia coli and Salmonella typhimurium were purchased from Ward’s Science (Rochester, NY, USA). An additional *E. coli* ΔtolC mutant was also used is this study; the mutation to the TolC multi-drug efflux pump decreases its activity and inhibitory activity against this strain provides evidence whether or not drug-efflux activity possessed by Gram-negative bacteria reduces the inhibitory activity of the extract [16]. Lipopolysaccharides (LPS) from *E. coli* were purchased from Sigma-Aldrich. A tetrazolium dye (WST-8) cell viability assay kit (CCK-8) was purchased from Dojindo Laboratories and an ELISA kit for TNF-α was purchased from eBioscience. All measurements from proliferation assays and ELISA experiments were performed using a 96-well microplate reader (Thermo-Fisher, Inc., Waltham, MA, USA). Two-way analysis of variance was performed using four replicate measurements of cell proliferation for each control and treatment group and three replicate ELISA measurements for each control and treatment group. 

### 2.2. Plant Material and Generation of Extracts

Flowers, leaves and stems of *T. lanatum* were collected in Malibu, California. Voucher specimens were deposited in the University of California, Los Angeles Herbarium at the Mildred E. Mathias Botanical Garden with the voucher specimen number LA212678. Flowers, leaves and stems (510 g) were cut into ~0.5–1 cm pieces and combined, then extracted three times with methanol (ACS grade) by percolation at room temperature. Methanol was removed by rotary and centrifugal evaporation, yielding a crude extract of the specimen (40.8 g). Portions of the extract were dissolved in 1 mL dimethyl sulfoxide (DMSO) to obtain solutions with desired concentrations for assays.

### 2.3. Immunomodulatory Assays

RAW 264.7 murine macrophage cells were cultured in Dulbecco’s Modified Eagle Medium (DMEM) with 10% fetal bovine serum (FBS) +1% antibiotic-antimycotic agent and incubated at 37 °C with 5% CO_2_. Because of their role in the response to infections, macrophages are typically treated (e.g., activated) with bacterial LPS to elicit a pro-inflammatory response [17]. LPS was dissolved in DMEM prior to application in cell cultures. For all assays cells were incubated for 16 h prior to treatments. Each assay included a negative control (0.01% *v*/*v* DMSO); no pretreatments were used. Cell proliferation was measured 24 h after treatment of cells using a tetrazolium dye (WST-8) cell viability assay kit. TNF-α concentrations were measured 12 and 24 h after treatments using an ELISA kit.

### 2.4. Antibacterial Disc Diffusion Assay

Bacterial cultures were diluted from overnight cultures to equivalent concentrations as determined from optical density measurements and then inoculated onto agar plates (nutrient agar or tryptic soy agar) using a cotton swab. A sterile paper disc dispenser was used to introduce filter paper discs onto the agar plates and 1.25 mg of the *T. lanatum* extract (10 μL of 125 mg/mL solution in DMSO) was added to each disc. Agar cultures were incubated at 37 °C for 18 h, except for *C. xerosis*, which was incubated for 40 h due its slow rate of growth. The diameter of each zone of inhibition was measured with a ruler. Ampicillin (1 μg/μL, aqueous) was used as a positive control and DMSO (10 µL) was used as a negative control. No zone of inhibition was observed surrounding discs treated with DMSO.

### 2.5. Chemical Analysis of T. lanatum Extract

The chemical composition of the *T. lanatum* extract was analyzed using liquid chromatography mass spectrometry (LC-MS). A Luna C18 5 μm 100 Å 250 × 10 mm HPLC column (Phenomonex, Torrance, CA, USA) was used for the analysis with a water:methanol gradient solvent system and a flow rate of 2 mL/min. A flow splitter was used to direct 500 μL/min of the eluent into the mass spectrometer while the remaining eluent was collected as fractions. The solvent program used for the analysis was 20% methanol for 5 min, a gradient of 20–100% methanol over 40 min, and finally a 100% methanol wash for 15 min. The injection amount of *T. lanatum* extract was 10 mg dissolved in 225 μL of methanol. The majority of distinct peaks eluted from 35–60 min. Unique compounds were identified in this region of the chromatogram using the ICIS integration algorithm in the Xcalibur 2.0.7 software (Thermo Fisher). The mass spectra for individual peaks were manually reviewed to identify spectra that contained [M + H^+^] molecular ion peaks corresponding to compounds previously identified in *T. lanatum*.

## 3. Results

### 3.1. Immunomodulatory Properties of T. lanatum Extract

**Figure 1:**
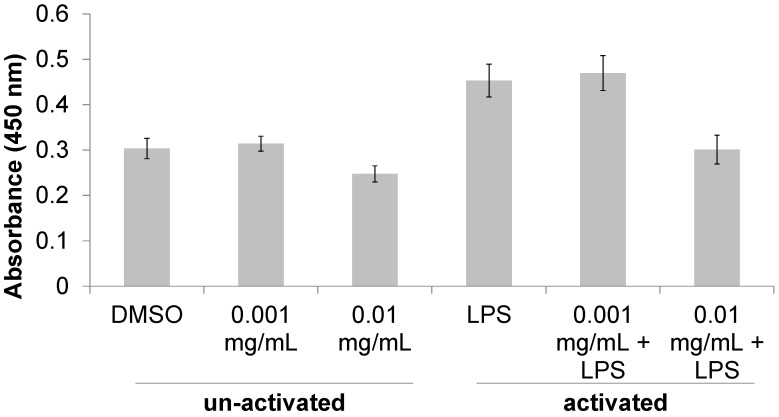
Cell proliferation activity of *T. lanatum* extract. The number of viable macrophage cells was determined by measuring the absorbance of cell cultures following the addition of tetrazolium dye, WST-8. Cells treated with DMSO (non-activated) were used as negative controls and cells treated with LPS (activated) were used to represent a pro-inflammatory response. Results of ANOVA suggest that activation of macrophages significantly affected cell proliferation (*P*_activation_ = 5.81 × 10^−9^) and that treatment with *T. lanatum* extract significantly altered cell proliferation (*P*_interaction_ = 3.16 × 10^−3^). Error bars represent ± one standard deviation from the mean response.

A methanolic extract was made from flowers, leaves and stems of *T. lanatum* specimens were collected in the Santa Monica Mountains in Malibu, CA. The methanolic extract was tested for immunomodulatory activity in RAW 264.7 murine macrophage cell assays. Proliferation of both activated and non-activated macrophages was measured 24 h after treatment with *T. lanatum* extract at concentrations of 0.001 mg/μL and 0.01 mg/μL (Figure 1). Activated macrophages proliferated 49% more than non-activated cells (*P* = 5.81 × 10^−9^), indicating that activation with LPS elicited a pro-inflammatory response. Treatment with 0.01 mg/mL *T. lanatum* extract caused a significant decrease in proliferation (*P* = 3.16 × 10^−3^) compared to LPS-treated cells and returned the activated macrophages to a proliferation level indistinguishable from that of the non-activated cells.

**Figure 2:**
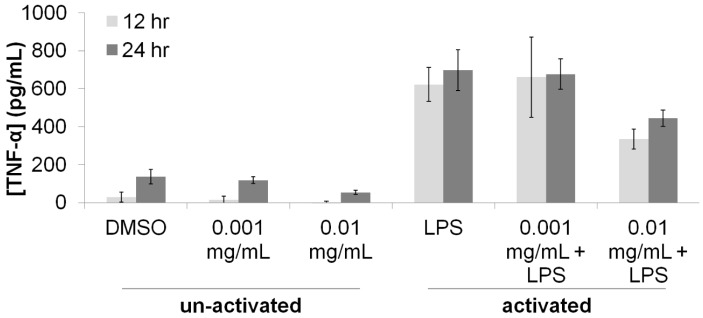
Immunomodulatory activity of *T. lanatum* extract. The amount of the pro-inflammatory cytokine TNF-α present in the supernatant of macrophage cultures was determined using ELISA at 12 and 24 h after treatments. Cells treated with DMSO (non-activated) were used as negative controls and cells treated with LPS (activated) were used to represent a pro-inflammatory response. Results from an ANOVA test suggest that activation of macrophages significantly alters [TNF-α] at both the 12-h (*P*_LPS-12h_ = 7.40 × 10^−8^) and 24-h (*P*_LPS-24h_ = 6.57 × 10^−10^) time-points. Treatment with 0.01 mg/mL *T. lanatum* extract significantly altered [TNF-α] at the 12-h (*P*_0.01mg/mL extract-12h_ = 1.29 × 10^−2^) and 24-h (*P*_0.01mg/mL extract-24h_ = 7.67 × 10^−4^) time-points. Error bars represent ± one standard deviation from the mean response.

To further investigate this putative reduction of the pro-inflammatory response of macrophages, the concentration of the pro-inflammatory cytokine TNF-α in the supernatant of macrophage cultures was measured using an immunoprecipitation assay (Figure 2). Activation of macrophages with LPS resulted in a 400% increase in the concentration TNF-α, providing evidence for a large pro-inflammatory response. Treatment of the activated macrophages with *T. lanatum* extract at a concentration of 0.001 mg/mL did not cause any changed in TNF-α concentrations, but treatment with 0.01 mg/mL resulted in a 200% reduction in TNF-α concentrations. These effects were reproducible at 12-h and at 24-h after treatment with LPS and *T. lanatum*. Results of ANOVA indicated that these effects were significant (*P*_LPS-12h_ = 7.40 × 10^−8^; *P*_LPS-24h_ = 6.57 × 10^−10^; *P*_0.01mg/mL extract-12h_ = 1.29 × 10^−2^; *P*_0.01mg/mL extract-24h_ = 7.67 × 10^−4^). 

### 3.2. Antibacterial Properties of T. lanatum Extract

Because of the reported traditional use of *T. lanatum* as a disinfectant, a panel of bacteria representing both Gram-negative and Gram-positive species was used to evaluate the anti-bacterial properties of the *T. lanatum* extract. An *Escherichia coli* ΔtolC mutant was also used to test whether or not drug-efflux activity in the Gram-negative bacteria would reduce the inhibitory activity of the extracts [16]; the reduced activity of TolC-dependent multidrug efflux pumps has been shown to be an effective method for identifying antimicrobial plant natural products [18,19]. The inhibition of bacterial growth was measured using a Kirby-Bauer disc diffusion assay [20]. Our results showed that treatment with *T. lanatum* extract inhibited the growth of Gram-positive bacteria but not Gram-negative bacteria in a dose-dependent manner (Table 1). However, growth of the *E. coli* ΔtolC mutant was inhibited by 1.25 mg of *T. lanatum* extract, suggesting that the resistance of Gram-negative bacteria to the anti-bacterial activity of *T. lanatum* may be due the drug-efflux ability of some of these bacterial species.

### 3.3. Chemical Analysis of T. lanatum Extract

**Figure 3:**
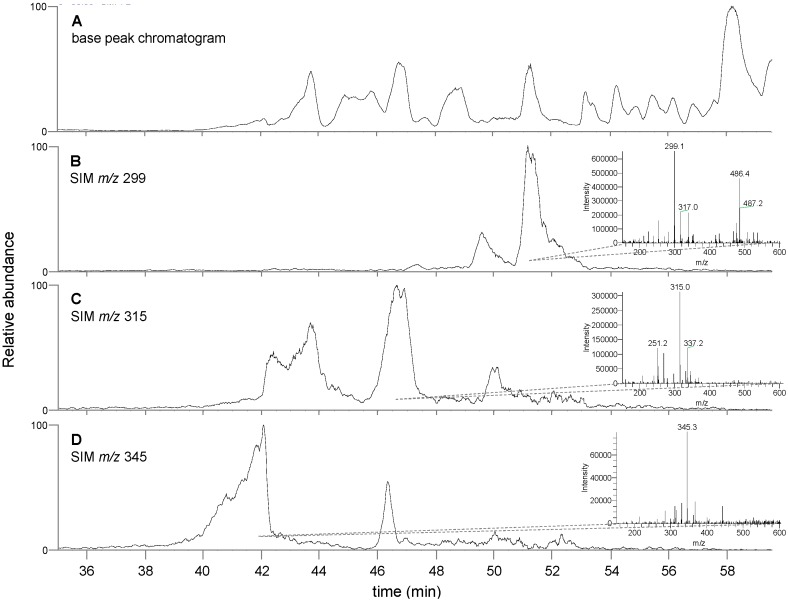
Analysis of chemical composition of *T. lanatum* extract using LC-MS. (**A**) Chromatogram generated by extracting the intensity of the base peak of the mass spectrum at each time point. Panels (**B**–**D**) represent the single ion chromatogram for the indicated *m*/*z* values which have been putatively matched the phenolic plant metabolites apigenin-7,4′-dimethyl ether ((**A**), *m*/*z* 299), either scutellarein-6,7-dimethyl ether or 6-hydroxygalangin 5,6-dimethyl ether ((**B**), *m*/*z* 315) and 6-hydroxy luteolin 6,7,4′-trimethyl ether ((**C**), *m*/*z* 345). Insets in panels (**B**–**D**) show an averaged mass spectrum for each region corresponding to the largest peak in each SIM chromatogram.

The chemical composition of the *T. lanatum* extract was analyzed using LC-MS. The base peak chromatogram of the extract was analyzed for *m*/*z* signatures characteristic of flavonoid aglycones that have previously been identified in exudates from *T. lanatum* [15]. Matching peak profiles were identified for the compounds apigenin-7,4′-dimethyl ether (*m*/*z* 299), either scutellarein-6,7-dimethyl ether or 6-hydroxygalangin 5,6-dimethyl ether (isomers, *m*/*z* 315) and 6-hydroxy luteolin 6,7,4′-trimethyl ether (*m*/*z* 345) (Figure 3). Identification of these compounds was based on matching low-resolution mass signatures.

## 4. Discussion

Traditional plant-based medicines remain an important resource for many people in the world, and in many locations these traditional medicines serve as a primary health care resource [21]. Unfortunately, many traditional plant-based medicines have never been experimentally evaluated and often the pharmacological basis of the medicinal properties of many of these plants is unknown [22,23]. The Chumash people were the indigenous inhabitants of Southern California for thousands of years prior to European colonization, and reportedly possessed an extensive number of medicinal remedies prepared from a wide variety of plants in California [4]. Very little is known about the chemical constituents of these Chumash medicinal plants or their pharmacological properties.

The results presented here provide experimental support for the traditional use of *T. lanatum* as a medicinal agent among the Chumash people. The reduction in proliferation and TNF-α production in the activated macrophages (Figure 1 and Figure 2) supports the hypothesis that the *T. lanatum* extract inhibits the pro-inflammatory response of activated macrophages [8,12]. The extract also exhibited anti-bacterial activity, although the doses used indicate the extract has less potency than the antibiotic ampicillin (Table 1). Additionally, the inhibitory activity of the extract against Gram positive bacteria but not Gram negative bacteria is consistent with a topical use for treating cuts or wounds, since many of the bacteria with pathogenic potential that are most often found on the skin are Gram positive [24,25].

The chemical analysis of the extract is also consistent with our bioassay results. Apigenin and galangin have both been repeatedly demonstrated to decrease the inflammatory response of LPS-activated macrophages [26,27,28,29]. Although the identification of specific flavonoids need to be confirmed with further analytical methods, the data is consistent with the observed bioactivity. A prior structure-activity relationship analysis supports the conclusion that flavonoids with the substitution pattern of these compounds match the expected pattern for anti-inflammatory activity [30]. Specifically, in the 2006 paper, the authors showed that the anti-inflammatory activity of flavonoids in rat macrophages is dependent on specific hydroxyl substitution patterns. Apigenin, a flavone with hydroxyl substitutions at the 5,7 and 4′ positions, was a potent inhibitor of PGE_2_ production in rat macrophages with an IC_50_ value 3.3 µM; the flavonone galangin, with hydroxyl substitutions at the 3, 5 and 7 positions exhibited an IC_50_ value of 14.3 µM. Although no methyl esters were tested in that study, the compounds identified in our analysis of the *T. lanatum* extract possess the same substitution patterns as the most active compounds reported by Takano-Ishikawa and colleagues. Apigenin-7,4′-dimethyl ether has the identical oxygen substitution pattern as apigenin, but with methoxy groups instead of hydroxyl groups at the 7 and 4′ positions. The compounds scutellarein-6,7-dimethyl ether (5,6,7,4′ substitutions) and 6-hydroxygalangin 5,6-dimethyl ether (3,5,6,7 substitutions) each possess substitution patterns that match the active compounds galangin (3,5,7 substitutions) and baicalein (5,6,7 substitutions, IC50 2.5 µM). The substitution pattern of 6-hydroxy luteolin 6,7,4′-trimethyl ether (5,6,7,3′,4′ substitutions) identified in our analysis is interesting because the closest analog in the report by Takano-Ishikawa and colleagues is luteolin (5,7,3′,4′ substitutions), which was not active in rat macrophages. It is possible that either this compound is not contributing to the activity reported in our macrophage assays, or that the addition of the substituent at the 6 position is sufficient to restore anti-inflammatory activity to this compound.

Complete validation of the traditional use of plants such as *T. lanatum* remains difficult due to the challenges associated with conducting meaningful human clinical trials. However, the results presented here provide both pharmacological and chemical support for the reported traditional use of *T. lanatum* by the Chumash for the treatment of cuts and wounds. It is reasonable to infer that antibacterial activity against Gram positive bacteria and anti-inflammatory activity in macrophages would be beneficial in preventing infection of cuts and wounds and promoting wound-healing.

## 5. Conclusions

Treatment of LPS activated mouse macrophages with *T. lanatum* extract caused a significant decrease in macrophage proliferation and a significant decrease in the production of TNF-α by the macrophage cells. Treatment with *T. lanatum* extract inhibited the growth of Gram-positive bacteria in a disc diffusion assay. Treatment of Gram-negative bacteria with *T. lanatum* extract did not inhibit bacterial growth, but this may be due to the activity of drug efflux pumps in these bacteria since the growth of *E. coli* ΔtolC was inhibited by the extract. Chemical analysis of the *T. lanatum* extract identified compounds that have previously been identified in *T. lanatum* and that have been reported to exhibit anti-inflammatory properties in macrophages. These results provide support for the traditional medicinal use of this plant by the Chumash people in Southern California and illustrate the potential scientific and medicinal value of further investigations of the molecular mechanisms of traditional medicines from America Indian groups.

## Figures and Tables

**Table 1:** Antibacterial activity of *T. lanatum* extract ^a^.

Bacterium	Gram (+/−)	Ampicillin (0.01 mg)	*T. lanatum* Extract		
			(1.25 mg)	(0.50 mg)	(0.20 mg)
*Corynebacterium xerosis*	+	53.0 ± 9.0	19.6 ± 1.9	17.7 ± 1.2	14.0 ± 1.2
*Enterococcus faecalis*	+	25.3 ± 0.3	11.6 ± 0.2	9.0 ± 0.3	no inhibition
*Bacillus subtilis*	+	27.3 ± 0.7	11.4 ± 0.8	9.7 ± 0.6	6.1 ± 3.3
*Staphylococcus epidermidis*	+	16.0 ± 0.7	11.1 ± 0.7	3.7 ± 4.3	no inhibition
*Bacillus megaterium*	+	23.8 ± 1.1	10.9 ± 0.7	9.0 ± 1.2	no inhibition
*Staphylococcus aureus*	+	31.0 ± 1.0	9.2 ± 0.4	no inhibition	no inhibition
*Escherichia coli *	−	15.1 ± 0.5	no inhibition	no inhibition	no inhibition
*Escherichia coli* Δ*tolC*	−	16.3 ± 0.3	12.3 ± 1.2	2.7 ± 2.7	no inhibition
*Salmonella typhimurium*	−	25.1 ± 1.6	no inhibition	no inhibition	no inhibition

^a^ Reported as diameter of zones of inhibition (mm).
